# Segmentation-model-based framework to detect aortic dissection on non-contrast CT images: a retrospective study

**DOI:** 10.1186/s13244-025-02098-z

**Published:** 2025-09-25

**Authors:** Qidong Wang, Shan Huang, Weifeng Pan, Zhan Feng, Lei Lv, Dongwei Guan, Zhiwen Yang, Yimin Huang, Wei Liu, Weiwei Shui, Mingliang Ying, Wenbo Xiao

**Affiliations:** 1 Department of Radiology, First Affiliated Hospital, School of Medicine, Zhejiang University, Hangzhou, China; 2 ShuKun Technology Co., Ltd., Jinhui Bd, Beijing, China; 3 Department of Radiology, Jinhua Municipal Central Hospital, Jinhua, China

**Keywords:** Aortic dissection, Non-contrast computed tomography, Deep learning.

## Abstract

**Objectives:**

To develop an automated deep learning framework for detecting aortic dissection (AD) and visualizing its morphology and extent on non-contrast CT (NCCT) images.

**Materials and methods:**

This retrospective study included patients who underwent aortic CTA from January 2021 to January 2023 at two tertiary hospitals. Demographic data, medical history, and CT scans were collected. A segmentation-based deep learning model was trained to identify true and false lumens on NCCT images, with performance evaluated on internal and external test sets. Segmentation accuracy was measured using the Dice coefficient, while the intraclass correlation coefficient (ICC) assessed consistency between predicted and ground-truth false lumen volumes. Receiver operating characteristic (ROC) analysis evaluated the model’s predictive performance.

**Results:**

Among 701 patients (median age, 53 years, IQR: 41–64, 486 males), data from Center 1 were split into training (439 cases: 318 non-AD, 121 AD) and internal test sets (106 cases: 77 non-AD, 29 AD) (8:2 ratio), while Center 2 served as the external test set (156 cases: 80 non-AD, 76 AD). The ICC for false lumen volume was 0.823 (95% CI: 0.750–0.880) internally and 0.823 (95% CI: 0.760–0.870) externally. The model achieved an AUC of 0.935 (95% CI: 0.894–0.968) in the external test set, with an optimal cutoff of 7649 mm^3^ yielding 88.2% sensitivity, 91.3% specificity, and 89.0% negative predictive value.

**Conclusions:**

The proposed deep learning framework accurately detects AD on NCCT and effectively visualizes its morphological features, demonstrating strong clinical potential.

**Critical relevance statement:**

This deep learning framework helps reduce the misdiagnosis of AD in emergencies with limited time. The satisfactory results of presenting true/false lumen on NCCT images benefit patients with contrast media contraindications and promote treatment decisions.

**Key Points:**

- False lumen volume was used as an indicator for AD.
- NCCT detects AD via this segmentation model.
- This framework enhances AD diagnosis in emergencies, reducing unnecessary contrast use.

**Graphical Abstract:**

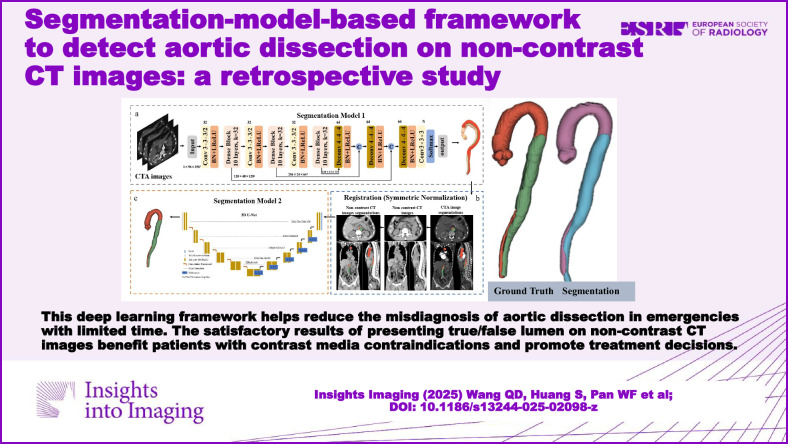

## Introduction

Aortic dissection (AD) is a life-threatening acute aortic syndrome with an estimated global incidence of 2–10 cases per 100,000 individuals annually, predominantly affecting males and patients with uncontrolled hypertension [1–5]. While computed tomography angiography (CTA) remains the gold standard for diagnosis, many patients initially undergo non-contrast CT (NCCT) during emergency evaluations, particularly those presenting with nonspecific symptoms. Furthermore, the use of iodinated contrast media in enhanced CT examinations is contraindicated in certain populations due to risks of severe allergic reactions and contrast-induced nephropathy [6]. Consequently, early detection of AD on NCCT images presents a critical clinical need. Although certain imaging features—such as aortic calcification displacement and intimal flap visualization—may suggest AD on NCCT [7, 8], their diagnostic sensitivity remains suboptimal. Alarmingly, approximately 30% of AD cases are initially misdiagnosed or experience delayed diagnosis [9, 10], contributing to a 48-h mortality rate of up to 30% if left untreated [8]. Improving AD detection on NCCT could significantly reduce misdiagnosis and delays, facilitating timely life-saving interventions.

In recent years, tremendous advances have been made in medical image-processing technology. Radiomics and deep learning algorithms have been investigated to make accurate diagnoses and prognostic predictions in many diseases, as in emergency fields like AD [11–13]. Radiomics signature at the level of trachea bifurcation on thoracic non-contrast images demonstrates an AUC of 90% in screening thoracic AD [14]. Deep learning algorithms based on Xception architecture, 3D MTGA, et al, all show good performance in classifying AD and non-AD with NCCT images [13, 15]. These studies need to be validated in large clinical populations, as noted.

In this study, we retrospectively included patients at two tertiary hospitals and developed a segmentation-model-based deep learning framework to detect AD and further provide its morphology and extent of involvement on NCCT images. In our study, false lumen volume, a quantitative result that is accurate, was used as an indication to predict the diagnosis of AD. As conceptualized, this framework is designed to accurately screen AD patients, particularly in emergency situations, especially for those with atypical symptoms and contrast media contraindications, while simultaneously generating the involved range for confirmed cases.

## Materials and methods

This is a retrospective study that includes real-world data sets from two tertiary hospitals. The Institutional Ethics Committees of the main center (center 1) gave their approval to the study (IIT20240706B), and the patients’ informed consent was waived.

### Patients

Patients who underwent aortic CTA examination in two centers between January 2021 and January 2023 were all initially included. The inclusion criteria were as follows: (1) The entire CTA examination should include both non-contrast and contrast-enhanced images. The thickness of the contrast-enhanced images should be less than 1 mm (no thickness requirement for NCCT). (2) No intra-aortic devices were present in the patients. The exclusion criteria were as follows: (1) Poor quality of the image, including poor contrast for contrast-enhanced images and images with heavy artifacts. (2) The patients have a history of aortic or heart surgery. All these included CTA images were reviewed by two radiologists with experience of more than 5 years (S.H. and Z.F.) with the inclusion and exclusion criteria. The diagnosis of AD was made according to the CTA images and the hospital records.

All included patients from center 1 were randomly divided into the training set and internal test set as a ratio of 8:2, and center 2 was designated as the external test set.

### CT images acquisition

All the CT examinations were performed using at least a 64-slice wide volume coverage CT scanner (GE, Philips, and Siemens). The scanning parameters of different CT scanners were demonstrated in the supplementary Table 1. For the enhanced imaging, 70–80 mL iodine contrast media (350 mgI/mL) were administered using a dual-head CT power injector via an antecubital vein at a rate of 4.5 mL/s, followed by 50 mL saline solution at the same rate. The bolus tracking technique was used to trigger the start of acquisition, and the scanning range covered the entire aorta course, beginning at the thoracic inlet and ending at the pubic symphysis.

### CT imaging process of the deep learning framework

#### Automatic true lumen and false lumen segmentation

**Fig. 1:**
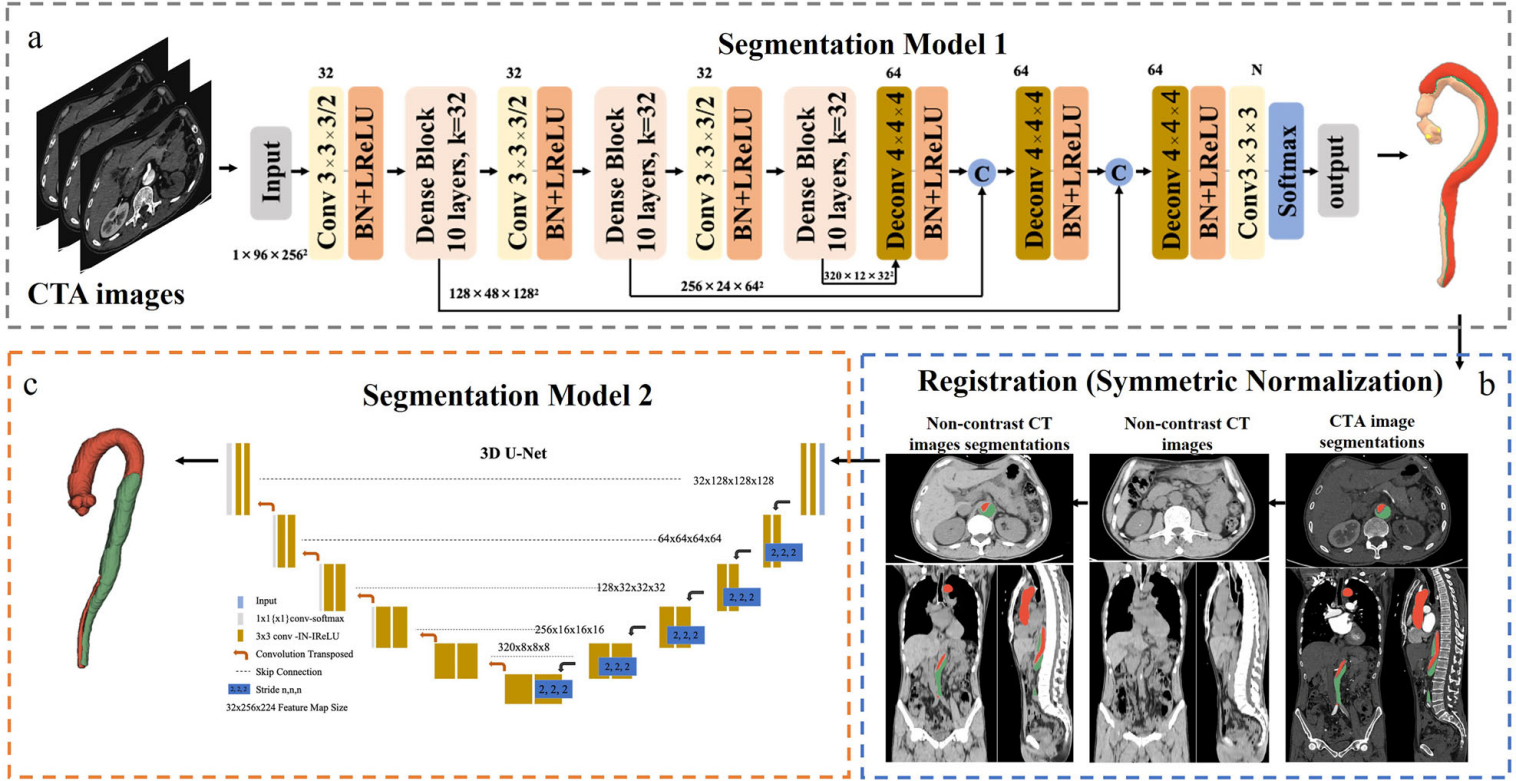
The pipeline of the deep learning framework. As shown in **a** (adapted from our previous work [16]), CTA images were input into segmentation model 1 to segment the aorta into the dual lumen. The masks of the true and false lumen were corrected by experienced radiologists. Corrected true and false lumen masks were used as ground truth for the next step segmentation. CTA and NCCT images were registered (**b**). Segmentation model 2 was applied to segment true and false lumens on NCCT images (**c**). And the segmented true/false lumen was compared with the standard true/false lumen

The automatic true lumen and false lumen segmentation algorithm can be found in our previous study [16]. CTA images are input into segmentation model 1 (Fig. 1). For AD cases, the true lumen and false lumen were segmented, respectively. For non-AD cases, the aorta was segmented and used as the true lumen in the subsequent process.

#### Manual segmentation correction

Segmentations of the true lumen and false lumen in the above steps were further reviewed and corrected by experienced radiologists in CTA images, and the corrected segmentations were used as ground truth masks for the subsequent model training. Manual segmentation correction was completed using medical image processing software Mimics 19.0 (Materialize).

#### Image registration of CTA and NCCT images

This study employed a non-rigid registration method based on symmetric normalization (SyN) to ensure the accurate alignment of the ground truth masks of the CTA images with the NCCT images (the detailed method for SyN was provided in the Supplementary Materials). The SyN non-rigid registration was implemented using the ANTs toolbox with its default parameter settings (including Mattes mutual information as the similarity metric and a multi-resolution iteration schedule of 40 × 20 × 10). Firstly, the images are globally aligned using a preliminary linear transformation. Following this, a multi-scale nonlinear optimization is performed using the SyN method, which enables the capture of complex deformations between the images. Thereafter, the derived deformation field is utilized in a nearest neighbor interpolation to transform the segmented image, ensuring that the registered anatomical structures maintain high consistency with the original image. This method demonstrably enhances the precision of alignment in multimodal image analysis.

#### Segmentation of true/false lumen on NCCT images

The three-dimensional version of the No New U-net (nnU-Net) architecture was utilized to differentiate between true lumen and false lumen in CT images. The nnU-Net is a deep learning-based segmentation method that is capable of automatically configuring and executing the entire segmentation process, encompassing pre-processing, data enhancement, model training, and post-processing. Furthermore, to ascertain the model’s resilience, we conducted a three-fold cross-validation, a validation approach that randomly partitions the dataset into three equal segments, with each segment serving as a test set and the remaining segment as a training set. This method permits the evaluation of the model’s performance on disparate subsets of data, thereby ensuring the reliability and generalizability of the results. The training was conducted using the default automatically generated hyperparameters of nnU-Net, which include a learning rate initialized to 0.01 and a polynomial decay strategy, a loss function that is the sum of the Dice loss and the Cross Entropy loss, and an optimizer that uses stochastic gradient descent (SGD).

#### The performance of the model in detecting AD with predicted false lumen volume

During the initial phase of the study, every scan was labeled as either ‘AD’ or ‘non-AD’ (ground truth). The false lumen volume of the model’s predicted mask, acquired through the previous segmentation step, was measured in cubic millimeters (mm^3^) and designated as the predicted false lumen volume. The predicted false lumen volume was utilized as an indicator for AD in this deep learning model. A receiver-operating-characteristic (ROC) curve was plotted to demonstrate the ability of the predicted false lumen volume alone to differentiate between ‘AD’ and ‘non-AD’ groups. An optimal classification threshold was determined by the objective of maximizing the difference between the true positive rate and the false positive rate. With this cutoff value, confusion matrices were made.

The whole process of the deep learning framework was demonstrated in Fig. 1.

#### Traditional image features evaluated in the external test set

In the external test set, we further evaluated the occurrence rate of traditional image features in AD patients by a radiologist (W.X.) with more than 25 years of experience, lacking knowledge of medical history, and with anonymous images. The image features include internal displacement of intimal calcification, high-attenuation crescent, linear high density in the aortic lumen, and thoracic aortic aneurysm. The definition of these features is based on the previous study [17]. In the previous study, the specificity for these four features almost reached 100%, which means the false positive rate almost reached 0. It is indicated that these features are unlikely to be found in non-AD patients, so we only evaluate the occurrence rate of these features in AD patients.

### Statistical analysis

The characteristics of patients in the training, internal test, and external test sets were summarized as median (interquartile range) for continuous variables, and number (percent) for categorical variables. Patient characteristics were compared across datasets using either the Kruskal-Wallis test for continuous non-normal distribution variables or one-way ANOVA analysis for continuous normal distribution variables. For category variables, the chi-square test was used. The Dice coefficient and intraclass correlation coefficient (ICC) were used to evaluate the segmentation consistency of the true/false lumen on NCCT images. The Bland–Altman plots were drawn visually. To evaluate the performance of the model in classifying cases into AD or non-AD with false lumen volume, ROC curves were plotted, and the area under the curve (AUC) was evaluated. An optimal cut-off value was determined by the objective of maximizing the difference between the true positive rate and the false positive rate, and confusion matrices were created. Performance indicators, including sensitivity, specificity, positive predictive value (PPV), and negative predictive value (NPV) were calculated. All these calculations were performed in Python (version 3.8), and the two-sided *p* value less than 0.05 was used.

## Results

### Patients characteristics

**Fig. 2:**
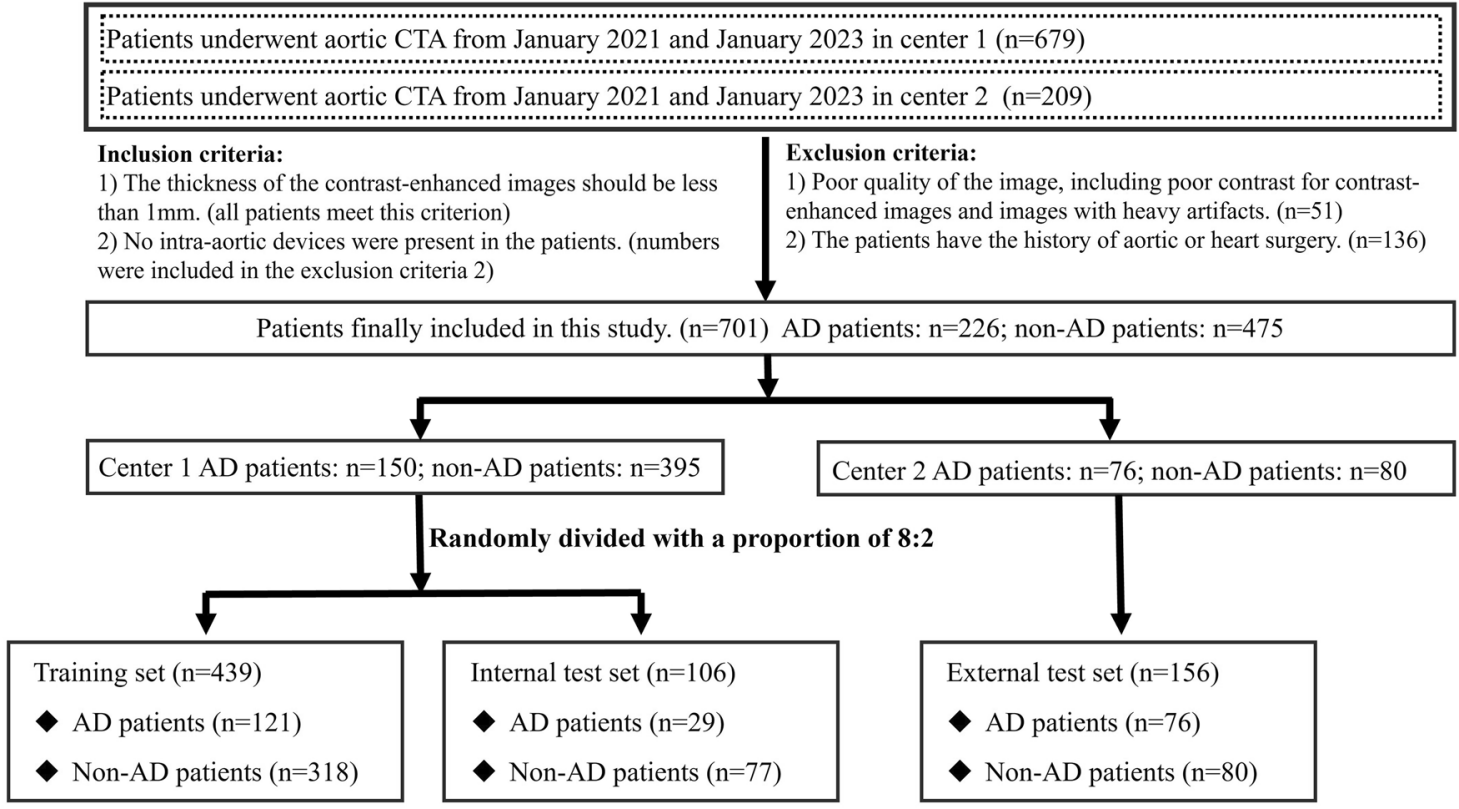
The flowchart for recruiting patients

A total of 888 patients (679 in center 1, 209 in center 2) were initially recruited. After a series of inclusion and exclusion criteria, 701 patients were finally included in the analysis. The flowchart for recruiting patients is presented in Fig. 2.

The patients in center 1 were divided into a training, internal test set with a ratio of 8:2, with 439 cases (318 non-AD/ 121 AD) in the training set and 106 cases (77 non-AD/29 AD) in the internal test set. Patients in the center 2 with 156 cases (80 non-AD/76 AD) were designated as the external test set. Patient characteristics are summarized in Table 1. The AD patients and non-AD patients are 54 (interquartile range [IQR]: 44–62), 56 (IQR: 43–66) years old in the training set, 52 (IQR: 48–60), 51 (IQR: 40–66) years old in the internal test set, and 52 (IQR: 43.5–68), 46.5 (IQR: 36.75–55) in the external test set (*p* = 0.066 across datasets). Males have a large distribution in all three datasets (62.5–86.3%, *p* = 0.437 across datasets). For all AD patients, 83 (68.6%) in the training set, 23 (79.3%) in the internal test set, and 61 (80.3%) in the external test set had chest/back pain when admission (*p* = 0.277 across datasets). 58 (47.9%), 18 (62.1%), 47 (61.8%) have a history of hypertension, respectively (*p* = 0.185 across datasets).

**Table 1:** Characteristics of included patients

Characteristics	Training set		Internal test set		External test set		*p* value
	AD patients (*n* = 121)	Non-AD patients (*n* = 318)	AD patients (*n* = 29)	Non-AD patients (*n* = 77)	AD patients (*n* = 76)	Non-AD patients (*n* = 80)	
Age, years	54 (44–62)	56 (43–66)	52 (48–60)	51 (40–66)	52 (43.5–68)	46.5 (36.75–55)	0.066
Sex (male)	91 (75.2%)	208 (65.4%)	25 (86.3%)	54 (70.1%)	58 (76.3%)	50 (62.5%)	0.437
Chest/back pain	83 (68.6%)	190 (59.7%)	23 (79.3%)	36 (46.7%)	61 (80.3%)	41 (51.3%)	0.277
History of hypertension	58 (47.9%)	104 (32.7%)	18 (62.1%)	28 (36.4%)	47 (61.8%)	22 (27.5%)	0.185

A continuous variable, such as age, was presented as median (interquartile range). Categorical variables, including sex, chest/back pain symptoms, and hypertension history, were presented as numbers (percent)

### The consistency of the segmentation model

**Fig. 3:**
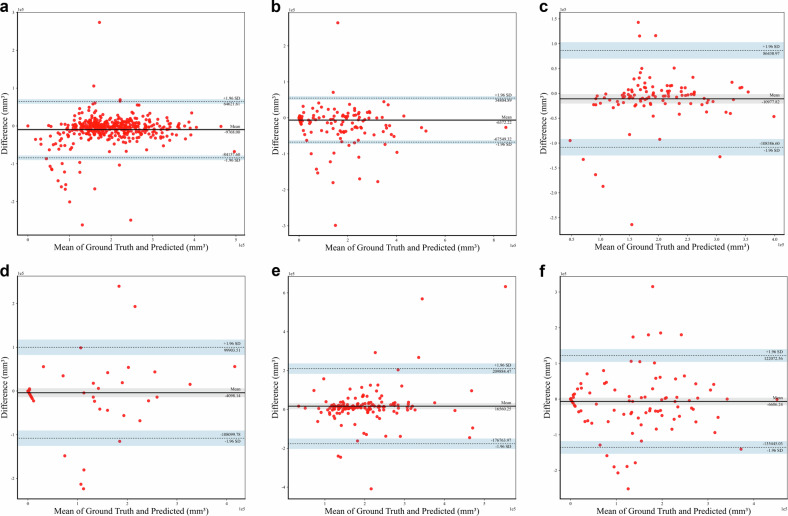
The Bland–Altman plots compare the predicted true/false lumen volume with the ground-truth true/false lumen volume. The Bland–Altman plots for the true lumen in the training (**a**), internal test (**c**), and external test set (**e**). The Bland–Altman plots for the false lumen in the training (**b**), internal test (**d**), and external test set (**f**). Difference refers to the predicted true/false, the ground-truth true/false lumen. Horizontal lines refer to the mean difference and 95% limits of agreement

We first segmented the true/false lumen on the arterial phase of the aortic CTA images, and then registered the mask on the corresponding NCCT images to obtain the standard true/false lumen on NCCT images. Following numerous iterations of training, we developed a nnU-Net model with robust segmentation performance to segment the predicted true/false lumen on NCCT images. The processing time for this model is about 1 min per case. The dice coefficients for the true lumen and the false lumen in the training set are 0.842 (IQR: 0.818–0.857), 0.707 (IQR: 0.655–0.752), in the internal test set are 0.797 (IQR: 0.757–0.827), 0.515 (IQR: 0.408–0.609), and in the external test set are 0.695 (IQR: 0.651–0.737), 0.603 (IQR: 0.547–0.651). The ICC for the false lumen volume is 0.957 (95% CI: 0.950–0.960) in the training set, 0.823 (95% CI: 0.750–0.880) in the internal test set, and 0.823 (95% CI: 0.760–0.870) in the external test set. The Bland–Altman plots comparing the predicted true/false lumen volume with the ground-truth true/false lumen volume are presented in Fig. 3a–f.

### The performance of the model in detecting AD with predicted false lumen volume

**Fig. 4:**
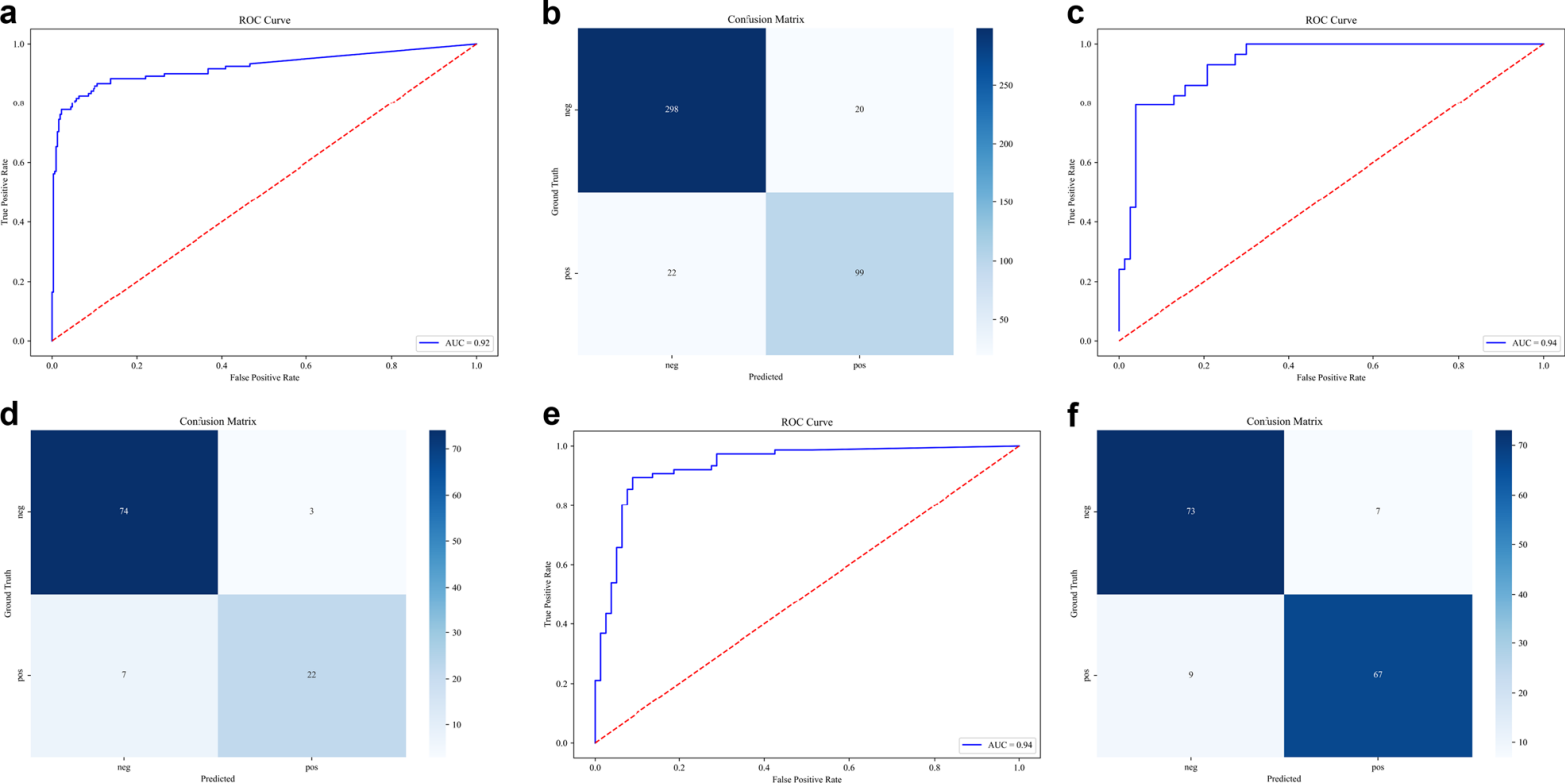
The ROC curve and the confusion matrix for the predicted false lumen volume. The ROC curve for the predicted false lumen volume and the confusion matrix in the training (**a**, **b**), internal test set (**c**, **d**), and external test set (**e**, **f**). ROC, receiver operating characteristic

The false lumen volume was used to indicate that if there is an AD. The false lumen volume value of 0 was referred to a non-AD case. To evaluate the performance of our model for classifying the case into AD or non-AD with predicted false lumen volume, ROC curves were plotted (Fig. 4a, c, e). The results demonstrate that the AUC for the training set, internal test set, and external test set are 0.921 (95% CI: 0.885–0.955), 0.938 (95% CI: 0.887–0.974), 0.935 (95% CI: 0.894–0.968), respectively. With the optimal cut-off value of 7649 mm^3^ for the volume of the false lumen in the training set, the sensitivity is 0.759, the specificity is 0.961, the PPV is 0.880, and the NPV is 0.871, respectively, in the internal test set. For the external test set, the specificity is 0.882, the specificity is 0.913, the PPV is 0.905, and the NPV is 0.890. In order to provide a more intuitive demonstration of the classification performance, confusion matrices were demonstrated in Fig. 4b, d, f. The confusion matrices provide a comprehensive representation of the classification outcomes, whereas the ROC curves elucidate the inherent trade-off between sensitivity and specificity. In general, the algorithm demonstrates satisfactory performance on the training, the internal test set, and the external test set. In addition, we conducted a subgroup analysis of the algorithm’s performance regarding complaints about chest/back pain. It is demonstrated that the algorithm performs satisfactorily in both patients with and without chest/back pain (AUC = 0.929 [95% CI: 0.887–0.969] and 0.906 [95% CI: 0.832–0.970] for patients with and without chest /back pain in the training set, 0.936 [95% CI: 0.864–0.990] and 0.862 [95% CI: 0.719–0.978] in the internal test set, 0.920 [95% CI: 0.853–0.974] and 0.950 [95% CI: 0.880–0.995] in the external test set). The ROC curves for this subgroup analysis are provided in the supplementary materials (Supplemental Figs. 1–3).

### Traditional image features evaluated in the external test set

In AD patients in the external test set, there is only a 22/76 (28.9%) occurrence rate for internal displacement of intimal calcification, 16/76 (21.1%) for high-attenuation crescent, 30/76 (39.5%) for linear high density in the aortic lumen, and 28/76 (36.8%) for thoracic aortic aneurysm. The percentage of cases with any of these four features is 52/76 (68.4%). It is noted that in all these 52 patients with any of these four features, our deep learning framework identified 46 cases. And in cases without these four features (24/76), there are also 21 cases that were detected as AD with the deep learning framework.

### Example cases for visualization

**Fig. 5:**
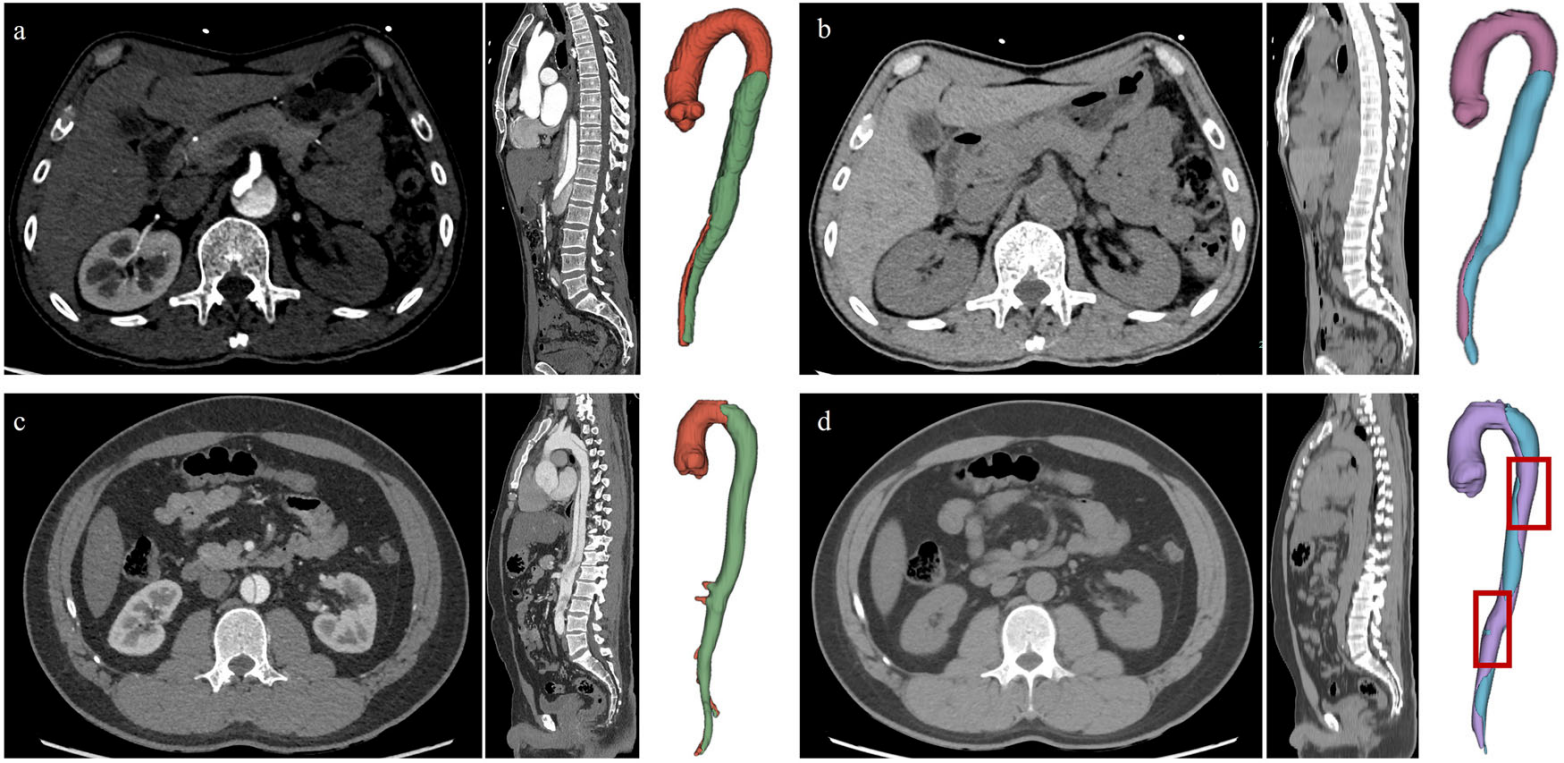
The examples of good and bad segmentation cases for AD. **a**, **b** A 64-year-old man, admitted to the hospital with back discomfort for one month. The CTA images and the 3D-volume reconstruction (VR) view of the aorta show the dissection of the aorta (**a**). The non-contrast images at the same level and the 3D-VR show good segmentation of true/false lumens on non-contrast images (**b**). The ground-truth false lumen volume is 107,282 mm^3^ and the predicted false lumen volume is 134,885 mm^3^. The Dice coefficient is 0.817. **c**, **d** A 33-year-old man, admitted to the emergency department with severe chest pain for 10 min. The 3D-VR of NCCT images presents a not-satisfactory segmentation (**d** compared with **c**). The standard false lumen volume is 155,658 mm^3^ and the predicted false lumen volume is 97,159 mm^3^. The Dice coefficient is 0.580

**Fig. 6:**
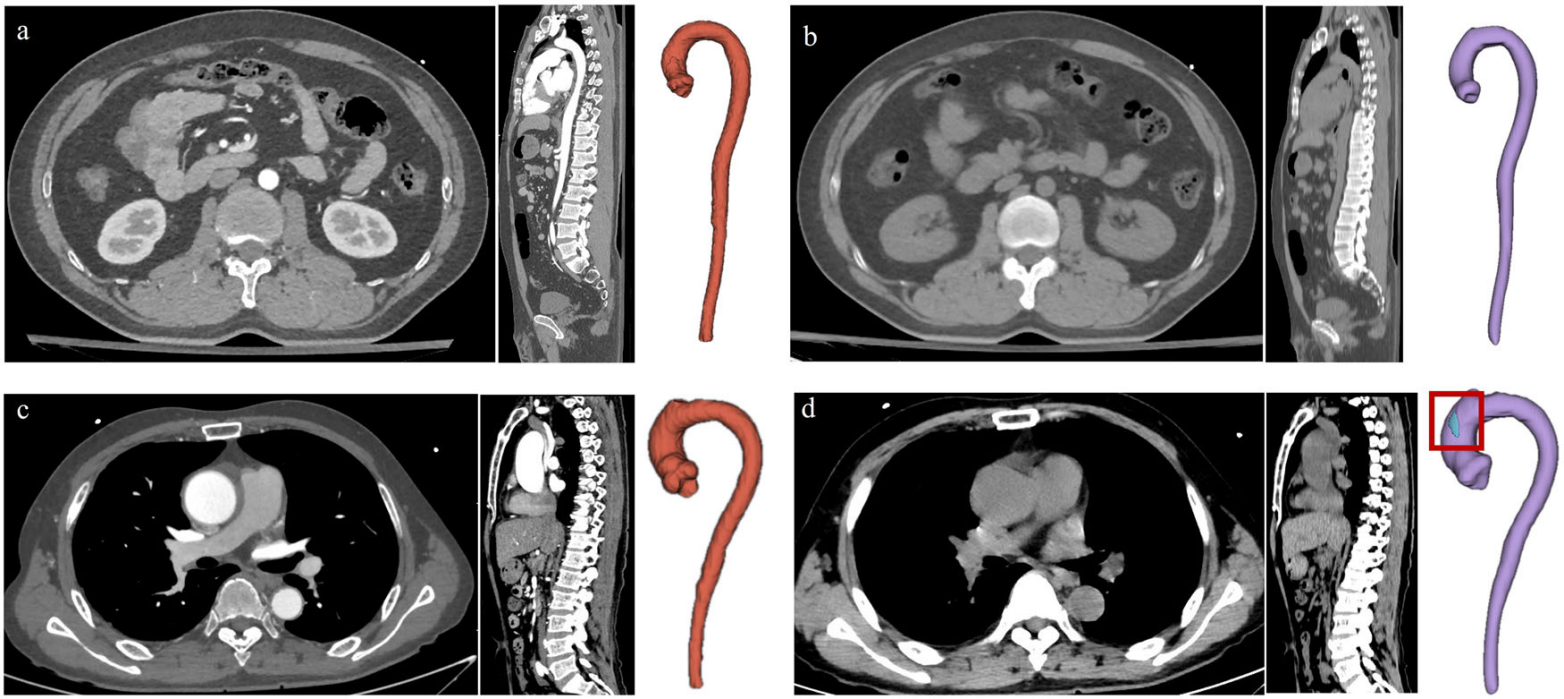
The examples of good and bad segmentation cases for non-dissection. **a**, **b** A 37-year-old man, admitted to the emergency department with back discomfort for several days. There is no positive finding in contrast-enhanced CTA images (**a**). The 3D-VR of the aorta in the right part shows that the false volume is 0 mm^3^ (**b**), which indicates that there is no AD. **c**, **d** A 53-year-old man, admitted to the emergency with a fall for 5 h. There is no positive finding on contrast-enhanced CTA images (**c**). However, the positive false lumen is detected with our deep learning framework (**d**)

Our deep-learning framework was conceptualized to generate the involved range for confirmed cases of AD. The Figs. 5a, b and 6a, b demonstrate the good performance of our model in detecting AD and non-AD cases, and provide direct visualization of the true and false lumen. However, several cases with poor performance also exist (Figs. 5c, d and 6c, d). Specific descriptions are present in the figure legends.

## Discussion

Radiological examination plays a key role in the diagnosis of AD, and CTA remains the gold standard for AD diagnosis. However, the contrast-enhanced CT is restricted to some conditions, and NCCT examinations are more widely adopted, especially in emergency situations and rural areas. The clinical focus has been on improving the sensitivity and accuracy of AD diagnosis on NCCT images. In this study, a robust deep-learning framework is utilized to segment true and false lumens on NCCT images. The AD is detected on NCCT images by using the false lumen volume as an indication. And the visualized segmentation reveals the morphology and extent of involvement of AD.

This study retrospectively collected data on 701 patients from two tertiary hospitals. The large sample size and real-world data promise the reliability of our results. The gender distribution of AD shows 299 (68.1%) males in the training set, 79 (74.5%) in the internal test set, and 108 (69.2%) in the external test set, which is consistent with the reported data that males are distributed nearly two-fold more than females in AD [4, 18]. The classic aortic pain is one of the most important clues in the first step of AD diagnosis, which is recommended by guidelines from the American Heart Association [19, 20]. Of all AD patients in this study, 68.6% in the training set, 79.3% in the internal test set, and 80.3% in the external test set experienced classic chest or back pain, which also means there are a number of patients that lack typical symptoms of AD, or take the aortic CTA examinations for other histories like trauma. Without this important information, the AD diagnosis becomes more challenging. Satisfactorily, the deep learning framework we developed in this study also shows good performance in these cases. This will undoubtedly help to detect AD with atypical symptoms in clinical practice, especially for emergencies.

There are several studies trying to apply automatic identification algorithms for AD [13–15, 21], and explainability may be a concern for methods like radiomics and end-to-end deep learning algorithms. In our study, we successfully segmented the true/false lumen on NCCT images and innovatively used the false lumen volume as an indication to diagnose the AD. The presentation of false lumen volume as an indication, results in more accurate quantitative results and a rationale that is understandable. As shown in the results, the optimal cutoff value is relatively small (7649 mm^3^), which indicates that the algorithm has the potential to figure out localized dissection of the aorta, though more validation data will be needed. The volume unit we used is mm^3^, which is a small unit where a slight error could have an impact on the results. This, to some degree, contributes to not having very high values for some model evaluation indicators. Through visualization of the segmented results of true and false lumen on NCCT images, the deep learning framework in our study also provides a direct visualization of AD morphology and range, which are beneficial for clinical purposes.

It is noteworthy that the deep learning framework we employed in this study is based on a segmentation model. nnU-Net, is a deep learning model widely used in medical CT image segmentation [22]. Based on excellent adaptability and simplified network design, the nnU-Net has higher segmentation efficiency, higher computational efficiency, and higher model generalization in medical CT image applications. However, nnU-Net also has some shortcomings, such as the large consumption of computing resources, and the problem of over-segmentation or under-segmentation when the contrast between blood vessels and surrounding tissues is low. Under-segmentation (Fig. 5c, d) and over-segmentation (Fig. 6c, d) can also be observed in this study. For the task of medical image segmentation, under- or over-segmentation is a very common phenomenon. In this study, segmentation of the true and false lumen on NCCT images is a difficult task since NCCT images have worse contrast, higher noise, and more image artifacts when compared to CTA images. As shown in Fig. 6c, d, the results of this over-segmentation may be attributed to the artifact in the aortic arch. This model has generally yielded good results, but there is still room for improvement in the future. For example, further labeling adjustment, parameter optimization of the model, sample size expansion, and improvements in the data enhancement strategy will be considered to improve the segmentation performance of this framework [12, 23, 24].

NCCT images indeed sometimes exhibit recognizable characteristics of AD, such as displacement of intimal calcification, high-attenuation crescent, and linear density in the aortic lumen. Nonetheless, these characteristics are limited in their ability to diagnose, or vulnerable to prompt the diagnosis of AD. As in the previous report, the sensitivity is between 59% and 77%, while the NPV is between 72% and 81% [17]. The sensitivity and NPV are the most important evaluation indicators for AD diagnosis. Misdiagnosis should be taken with great caution. In our external test set, each traditional image feature has a low occurrence rate of 21.1–39.5%. Moreover, in clinical emergency conditions, these subtle manifestations are prone to being ignored in the rush of time. In cases without traditional image features, the deep learning framework also detects the majority of cases (21/24).

Some limitations are also present in the study. Selection bias is unavoidable due to its retrospective nature, and the results need more validation to generalize their application. Second, the NCCT images we employed in this study are 3-mm-thick or 5-mm-thick slices. Thin slices that thickness-match the contrast-enhanced CTA images may yield better results with the segmentation model. However, it is noticeable that in clinical work, 5-mm-thickness images are commonly used for routine chest or abdominal CT exams. A segmentation model that is suitable for thick images may have a greater clinical potential.

In conclusion, the deep learning framework we developed in this study could accurately screen AD cases, even in patients with atypical symptoms and without traditional AD CT findings, thereby greatly reducing the misdiagnosis. Moreover, the visualization of true/false lumens on NCCT images could reveal the morphology and extent of AD involvement, which suggests the potential involved anatomical structures and organs. The described clinical utility is particularly pronounced in patients who cannot safely receive contrast agents and patients with atypical symptoms. In addition, by screening out AD-negative patients, this deep learning framework can effectively decrease the use of contrast agents, enhancing patient safety.

## Supplementary information

ELECTRONIC SUPPLEMENTARY MATERIAL

## Data Availability

The datasets used and analyzed during the current study are available from the corresponding author on reasonable request.

## Abbreviations

AD: Aortic dissection
AUC: Area under the curve
CTA: Computed tomography angiography
ICC: Intraclass correlation coefficient
NCCT: Non-contrast CT images
NPV: Negative predictive value
PPV: Positive predictive value
ROC: Receiver operating characteristic
SyN: Symmetric normalization
VR: Volume rendering
