# Supplementary material for: Segmentation-model-based framework to detect aortic dissection on non-contrast CT images: a retrospective study
Source: Insights Imaging. 2025 Sep 25;16:207. doi: 10.1186/s13244-025-02098-z (PMC12463791; doi:10.1186/s13244-025-02098-z)
Supplement: Supplementary file 1 — ELECTRONIC SUPPLEMENTARY MATERIAL [file 13244_2025_2098_MOESM1_ESM.pdf]

Segmentation-model-based framework to detect aortic dissection on non-contrast CT images: a retrospective study.

ELECTRONIC SUPPLEMENTARY MATERIAL

The detailed explanation for Symmetric Normalization (SyN)

Overview of the SyN Method:

Symmetric Normalization (SyN) is an advanced image registration algorithm designed to align images from different modalities or time points by optimizing the similarity between them. It is particularly useful for medical images where precise alignment of anatomical structures is crucial. The SyN method is part of the Advanced Normalization Tools (ANTs) suite, a collection of state-of-the-art image registration tools.

Steps Involved in the SyN Method:

Initialization: The process begins with the selection of a fixed image (the target) and a moving image (the source) that needs to be aligned to the fixed image. In our case, the fixed image is the NCCT image, and the moving image is the CTA image.

Similarity Metric: The SyN method uses a similarity metric to evaluate how well the moving image matches the fixed image. Common metrics include Mutual Information (MI), Cross-Correlation (CC), and Mean Squared Error (MSE). For our application, Mutual Information is often used due to its robustness in aligning images from different modalities.

Transformation Model: The SyN method employs a diffeomorphic transformation model, which ensures that the transformation between the moving and fixed images is invertible and topology-preserving. This is crucial for maintaining the anatomical integrity of the structures during the registration process.

Optimization: The registration process involves iteratively optimizing the transformation parameters to maximize the similarity metric. The SyN method uses a multi-resolution approach, starting with a coarse alignment and progressively refining it to achieve high precision.

Regularization: To prevent overfitting and ensure smooth transformations, the SyN method incorporates regularization techniques. These techniques penalize large deformations, ensuring that the transformations are anatomically plausible.

Application in Our Methodology:

Segmentation Mask Generation: Segmentation masks are generated from the CTA images using advanced segmentation algorithms. These masks accurately delineate the vascular structures and other relevant anatomical regions.

Registration Process: The CTA image (with segmentation masks) is registered to the NCCT image using the SyN method. The transformation parameters obtained from this process are then applied to the segmentation masks, effectively warping them to align with the NCCT image.

Ground Truth Alignment: The aligned segmentation masks serve as the ground truth for the NCCT images. This allows us to train and validate our segmentation models on NCCT images with high accuracy, leveraging the detailed information from the CTA-derived masks.

For readers who may not be familiar with the SyN method, we provide the following supplementary materials to aid understanding:

ANTs Documentation: Detailed documentation of the ANTs suite, including the SyN method, can be found at ANTs Documentation (https://github.com/ANTsX/ANTs/wiki).

Tutorial Videos: Step-by-step tutorial videos demonstrating the use of the SyN method for image registration are available on the ANTs YouTube channel.

Example Code: Example code snippets and Jupyter notebooks illustrating the implementation of the SyN method in Python and R can be accessed through the ANTs GitHub repository.

Supplemental table 1. CT parameters applied in this study

CT vendor	CT model name	Slice thickness/increment (NCCT-CTA)	Detector collimation	Matrix	KV	mAs
**SIEMENS**	SOMATOM Force	5.0/5.0-0.6/0.6mm	2*96*0.6mm	512×512	100	CareDose 4D
**SIEMENS**	SOMATOM go.Fit	5.0/5.0-1.0/0.6mm	64*0.6 mm	512×512	120	CareDose 4D
**Philips**	iCT 256	3.0/3.0-1.0/0.625mm	128*0.625mm	512×512	100	Dose Right
**Philips**	Brilliance 64	3.0/3.0-1.0/0.625mm	64*0.625mm	512×512	120	Dose Right
**GE**	LightSpeed VCT	5.0/5.0-0.625/0.625mm	64*0.625mm	512×512	120	Smart mA
**GE**	Revolution Frontier	5.0/5.0-0.625/0.625mm	64*0.625mm	512×512	100	Smart mA
**GE**	Revolution CT	5.0/5.0-0.625/0.625mm	256*0.625mm	512×512	120	Smart mA

The ROC curves for a subgroup analysis of the algorithm's performance regarding complaints about chest/back pain.

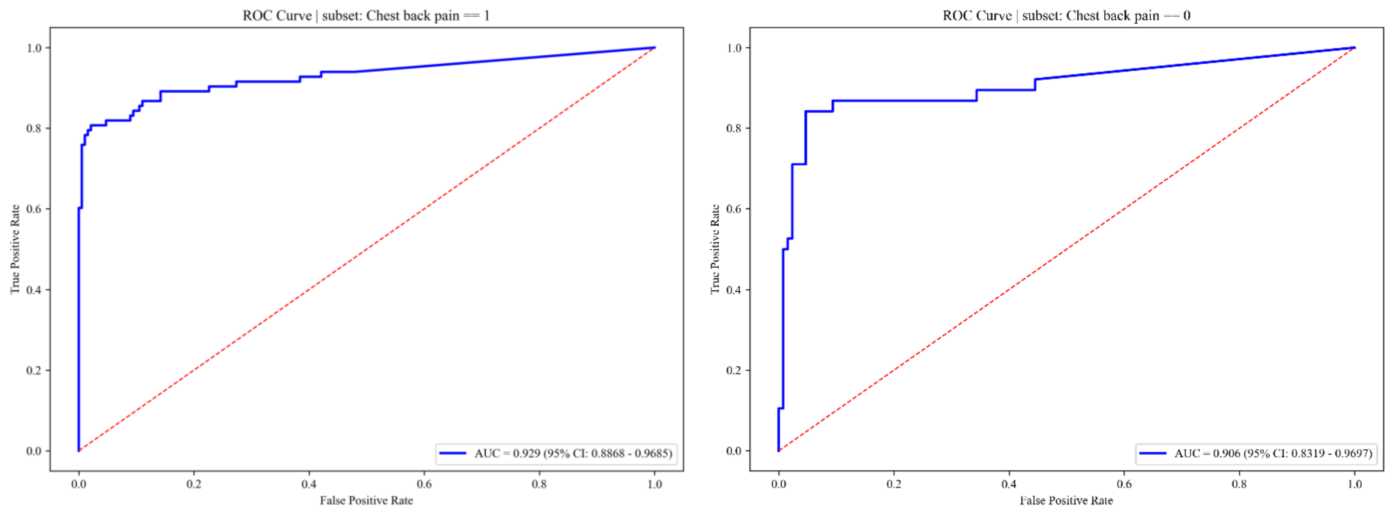

Supplemental Figure 1: the ROC curves for patients with (chest back pain = 1) and without (chest back pain = 0) in the training set.

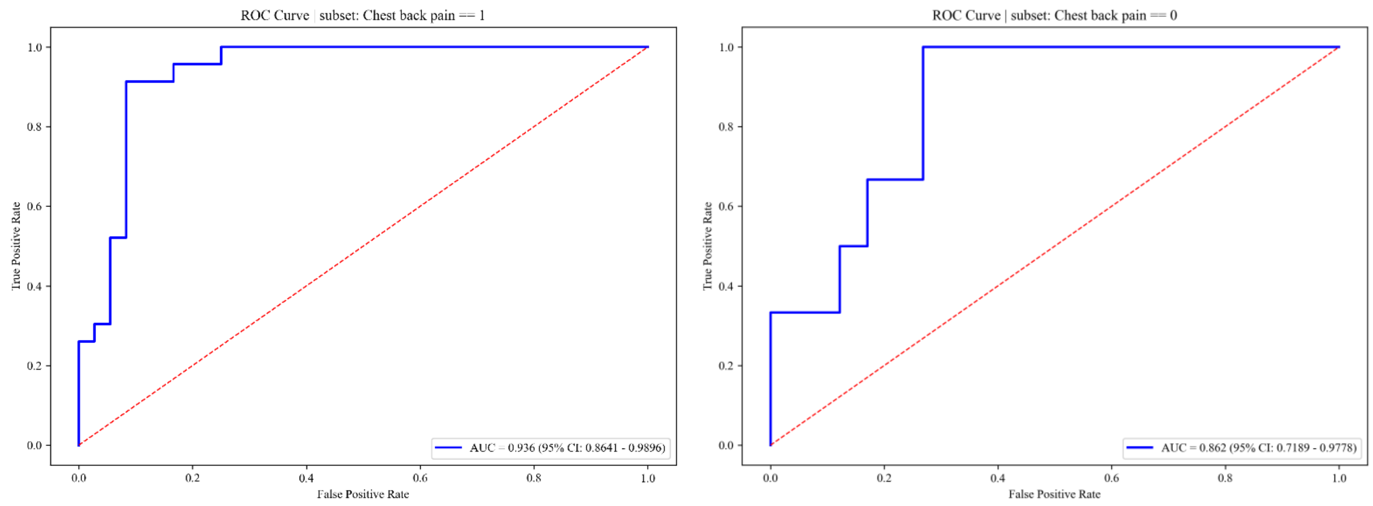

Supplemental Figure 2: the ROC curves for patients with (chest back pain = 1) and without (chest back pain = 0) in the internal test set.

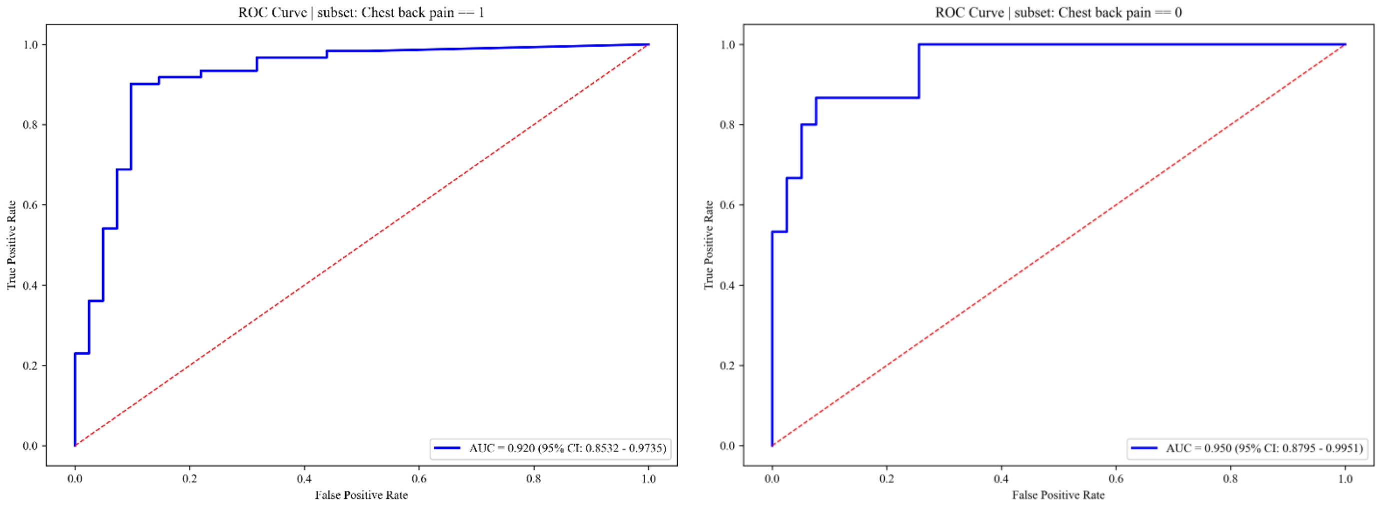

Supplemental Figure 3: the ROC curves for patients with (chest back pain = 1) and without (chest back pain = 0) in the external test set.
